# Activated platelet-rich plasma improves adipose-derived stem cell transplantation efficiency in injured articular cartilage

**DOI:** 10.1186/scrt277

**Published:** 2013-08-01

**Authors:** Phuc Van Pham, Khanh Hong-Thien Bui, Dat Quoc Ngo, Ngoc Bich Vu, Nhung Hai Truong, Nhan Lu-Chinh Phan, Dung Minh Le, Triet Dinh Duong, Thanh Duc Nguyen, Vien Tuong Le, Ngoc Kim Phan

**Affiliations:** 1 Laboratory of Stem Cell Research and Application, University of Science, Vietnam National University, 227 Nguyen Van Cu, District 5, Ho Chi Minh City, Vietnam; 2 University of Medical Center, Ho Chi Minh University of Medicine and Pharmacy, 215 Hong Bang, District 5, Ho Chi Minh City, Vietnam; 3 Department of Pathology, University of Medicine and Pharmacy, 217 Hong Bang, District 5, Ho Chi Minh City, Vietnam

**Keywords:** Adipose tissue-derived stem cells, Articular cartilage injury, Joint failure, Mesenchymal stem cells, Osteoarthritis, Platelet-rich plasma

## Abstract

**Introduction:**

Adipose-derived stem cells (ADSCs) have been isolated, expanded, and applied in the treatment of many diseases. ADSCs have also been used to treat injured articular cartilage. However, there is controversy regarding the treatment efficiency. We considered that ADSC transplantation with activated platelet-rich plasma (PRP) may improve injured articular cartilage compared with that of ADSC transplantation alone. In this study, we determined the role of PRP in ADSC transplantation to improve the treatment efficiency.

**Methods:**

ADSCs were isolated and expanded from human adipose tissue. PRP was collected and activated from human peripheral blood. The effects of PRP were evaluated *in vitro* and in ADSC transplantation *in vivo*. *In vitro*, the effects of PRP on ADSC proliferation, differentiation into chondrogenic cells, and inhibition of angiogenic factors were investigated at three concentrations of PRP (10%, 15% and 20%). *In vivo*, ADSCs pretreated with or without PRP were transplanted into murine models of injured articular cartilage.

**Results:**

PRP promoted ADSC proliferation and differentiation into chondrogenic cells that strongly expressed collagen II, Sox9 and aggrecan. Moreover, PRP inhibited expression of the angiogenic factor vascular endothelial growth factor. As a result, PRP-pretreated ADSCs improved healing of injured articular cartilage in murine models compared with that of untreated ADSCs.

**Conclusion:**

Pretreatment of ADSCs with PRP is a simple method to efficiently apply ADSCs in cartilage regeneration. This study provides an important step toward the use of autologous ADSCs in the treatment of injured articular cartilage.

## Introduction

Platelet-rich plasma (PRP) has been widely used across many clinical fields, especially for skincare and orthopedics. PRP contains at least seven growth factors including epidermal growth factor, platelet-derived growth factor, transforming growth factor-beta, vascular endothelial growth factor (VEGF), fibroblast growth factor, insulin-like growth factor, and keratinocyte growth factor. The therapeutic effect of PRP occurs because of the high concentration of these growth factors compared with that in normal plasma [1,2]. Many of these growth factors have important roles in wound healing and tissue regeneration. PRP stimulates the expression of type I collagen and matrix metalloproteinase-1 in dermal fibroblasts [3], and increases the expression of G_1_ cycle regulators, type I collagen, and matrix metalloproteinase-1 to accelerate wound healing [4].

In animal models, intra-articular PRP injection influences cartilage regeneration in all severities of rabbit knee osteoarthritis [5]. In a porcine model, PRP attenuates arthritic changes as assessed histologically and based on protein synthesis of typical inflammatory mediators in the synovial membrane and cartilage [6]. Clinically, PRP can repair cartilage with focal chondral defects. Siclari and colleagues performed this experiment on 52 patients (mean age: 44 years) with focal chondral defects in radiologically confirmed nondegenerative or degenerative knees [7]. Defects were coated with PRP-immersed polymer-based implant. Compared with the baseline and 3-month follow-up, the results showed that the Knee injury and Osteoarthritis Outcome Score showed clinically meaningful and significant improvement in all subcategories. Histological analysis of biopsied tissue showed hyaline-like to hyaline cartilage repair tissue that was enriched with cells showing a chondrocyte morphology, proteoglycans, and type II collagen (col-II) [7]. PRP injection with arthroscopic microfracture also improves early osteoarthritic knees with cartilage lesions in 40-year-old to 50-year-old patients, and the indication of this technique could be extended to 50-year-old patients [8]. In addition, PRP injection significantly improves the Visual Analog Scale for Pain score and the International Knee Documentation Committee score [9,10]. In a recent study with a larger patient cohort (120 patients), Spakova and colleagues showed that autologous PRP injection is an effective and safe method for the treatment of the initial stages of knee osteoarthritis [11]. In this research, 120 patients with Grade 1, Grade 2, or Grade 3 osteoarthritis according to the Kellgren and Lawrence grading scale were enrolled. Patients were treated using three intra-articular applications of PRP. Statistically significantly better results in the Western Ontario and McMaster Universities Osteoarthritis Index and the Numeric Rating Scale scores were recorded patients who received PRP injections after 3-month and 6-month follow-up.

Stem cells from adipose tissue were isolated and differentiated *in vitro* into adipogenic, chondrogenic, myogenic, and osteogenic cells in the presence of specific induction factors [12]. These cells are termed adipose-derived stem cells (ADSCs). ADSCs express surface markers as CD44, CD73, CD90, and CD105, but are negative for CD14, CD34, and CD45 [13-16]. This profile is similar to that of mesenchymal stem cells (MSCs) that have been suggested by Dominici and colleagues [17]. Compared with MSCs from bone marrow and umbilical cord blood, MSCs from adipose tissue have many advantages [18]. ADSCs are considered a suitable autologous cell source. Moreover, ADSCs have been used to treat many diseases such as liver fibrosis [19], nerve defects [20-22], ischemia [23,24], skeletal muscle injury [25], passive chronic immune thrombocytopenia [26], and infarcted myocardium [27] in animals; and systemic sclerosis in human [28,29].

ADSCs have been extensively investigated in preclinical studies for the treatment of cartilage injuries and osteoarthritis in animal models including dogs [30-32], rabbits [33], horses [34], rats [35], mice [36-38], and goats [39]. In a recent study, Xie and colleagues showed that ADSC-seeded PRP constructs develop into functional chondrocytes that secrete cartilaginous matrix in rabbits at 9 weeks post implantation [40]. These studies show evidence of functional improvement, especially scores for lameness, pain, and range of motion compared with control dogs [30-32], prevention of osteoarthritis and repair of defects in rabbit [33], upregulation of glycosaminoglycans as well as col-II to promote osteochondral repair and osteoarthritis prevention in rat [35], and protection against cartilage damage [36] as well as anti-inflammatory and chondroprotective effects [37] in mice following ADSC transplantation. These results have prompted human clinical trials for the treatment of osteoarthritis.

For example, Pak showed significant positive changes in all patients transplanted with ADSCs [41]. Various phase I and phase II clinical trials using ADSCs have been undertaken for osteoarthritis or degenerative cartilage (NCT01300598, NCT01585857 and NCT01399749). More importantly, in one clinical trial 18 patients underwent ADSC and PRP transplantation. The results of this study showed that intra-articular injection of ADSCs and PRP is effective for reducing pain and improving knee function in patients being treated for knee osteoarthritis [42].

In another study, however, ADSCs were considered to inhibit cartilage regeneration. This conclusion was drawn from experiments of ADSC transplantation in rats. This study showed that ADSCs highly express and secrete VEGF-A into the culture supernatant. The supernatant inhibits chondrocyte proliferation, reduces Sox9, alcan, and col-II mRNA levels, reduces proteoglycan synthesis, and increases apoptosis. ADSCs have been implanted in 1 mm noncritical hyaline cartilage defects *in vivo*, and showed inhibition of cartilage regeneration by radiographic and equilibrium partitioning of an ionic contrast agent via micro-computed tomography imaging. Histology revealed that defects with ADSCs had no tissue ingrowth from the edges of the defect [43].

Based on the above results, we considered that ADSC transplantation in combination with PRP might improve the efficiency of injured articular cartilage treatment. We theorized that PRP affects ADSC proliferation and differentiation, especially chondrogenic differentiation. This study therefore aimed to evaluate the effects of PRP on ADSC proliferation and differentiation into chondrocytes *in vitro*, and cartilage formation *in vivo*.

## Materials and methods

### Isolation of stromal vascular fraction cells from adipose tissue

Stromal cells were first isolated from the abdominal adipose tissue of 10 consenting healthy donors. From each patient, approximately 40 to 80 ml lipoaspirate was collected in two 50 ml sterile syringes. All procedures and manipulations were approved by our Institutional Ethical Committee (Laboratory of Stem Cell Research and Application, University of Science, Vietnam National University, Ho Chi Minh City, Vietnam) and the Hospital Ethical Committee (Ho Chi Minh City Medicine and Pharmacy University Hospital, Ho Chi Minh City, Vietnam). The syringes were stored in a sterile box at 2 to 8°C and immediately transferred to the laboratory. The stromal vascular fraction (SVF) was isolated using an ADSC Extraction kit (GeneWorld, Ho Chi Minh City, Vietnam) according to the manufacturer’s instructions. Briefly, 80 ml lipoaspirate was placed into a sterile disposable 250 ml conical centrifuge tube (2602A43; Corning 836, North Street Building, Tewksbury, MA 01876, USA). The adipose tissue was washed twice in PBS by centrifugation at 400 × *g* for 5 minutes at room temperature. Next, the adipose tissue was digested using the SuperExtract Solution (1.5 mg collagenase/mg adipose tissue) at 37°C for 30 minutes with agitation at 5-minute intervals. The suspension was centrifuged at 800 × *g* for 10 minutes, and the SVF was obtained as a pellet. The pellet was washed twice with PBS to remove any residual enzyme, and resuspended in PBS to determine the cell quantity and viability using an automatic cell counter (NucleoCounter; Chemometec, Gydevang 43, DK-3450 Allerod, Denmark).

### Platelet-rich plasma preparation

Human PRP was derived from the peripheral blood of the same donor as the adipose tissue using a New-PRP Pro Kit (GeneWorld) according to the manufacturer’s guidelines. Briefly, 20 ml peripheral blood was collected into vacuum tubes and centrifuged at 800 × *g* for 10 minutes. The plasma fraction was collected and centrifuged at 1000 × *g* for 5 minutes to obtain a platelet pellet. Most of the plasma was then removed, leaving 3 ml plasma to resuspend the platelets. This preparation was inactivated PRP. Finally, PRP was activated by activating tubes containing 100 μl of 20% CaCl_2_.

### Adipose-derived stem cell culture

SVF cells were cultured to expand the number of ADSCs. SVF cells were cultured in DMEM/F12 (Sigma-Aldrich, St Louis, MO, USA) containing 1× antibiotic–mycotic and 10% fetal bovine serum (FBS; Sigma-Aldrich) at 37°C with 5% CO_2_. The medium was changed twice per week. At 70 to 80% confluence, the cells were subcultured using 0.25% trypsin/ethylenediamine tetraacetic acid (GeneWorld).

### Cell proliferation assay

A total of 5 × 10^3^ ADSCs per well were cultured in 96-well plates in 100 μl DMEM/F12 containing 10% PRP, 15% PRP, 20% PRP, or 10% FBS as the control.

Twenty microliters of MTT (5 g/l; Sigma-Aldrich) was added to each well, followed by incubation for 4 hours and then addition of 150 μl DMSO/well (Sigma-Aldrich). Plates were then agitated for 10 minutes until the crystals dissolved completely. Absorption values were measured at a wavelength of 490 nm and a reference wavelength of 630 nm using a DTX 880 microplate reader (Beckman Coulter, Krefeld, Germany).

### Immunophenotyping

Third-passage ADSCs were examined for their immunophenotype by flow cytometry according to previously published protocols [44]. Briefly, cells were washed twice in Dulbecco’s PBS containing 1% BSA (Sigma-Aldrich). Cells were stained for 30 minutes at 4°C with anti-CD14-fluorescein isothiocyanate, anti-CD34-fluorescein isothiocyanate, anti-CD44-phycoerythrin, anti-CD45-fluorescein isothiocyanate, anti-CD90-phycoerythrin, or anti-CD105-fluorescein isothiocyanate mAb (BD Biosciences, Franklin Lakes, NJ, USA). Stained cells were analyzed by a FACSCalibur flow cytometer (BD Biosciences). Isotype controls were used for all analyses.

### Gene expression analysis

Third-passage ADSCs were evaluated for the effects of PRP on their proliferation and differentiation. ADSCs were cultured in six-well plates at 1 × 10^5^ cells/well in DMEM/F12 with 10% FBS and 1% antibiotic–mycotic for 24 hours. The medium was then replaced with DMEM/F12 with 1% antibiotic–mycotic and 10% PRP, 15% PRP, 20% PRP, or 10% FBS as the control. ADSCs were cultured under these conditions for 1 week with two medium changes per week. ADSCs were then isolated to evaluate their gene expression.

Total RNA was extracted as described elsewhere [44]. RNA was precipitated with 500 μl isopropyl alcohol at room temperature for 10 minutes. ADSCs were analyzed for the expression of chondrogenic markers including col-II, Sox9, and aggrecan. Real-time RT-PCR was performed with an Eppendorf gradient S thermal Cycler (Eppendorf-AG, Hamburg, Germany). The reaction mixture (25 μl) contained 10 mM Tris–HCl, pH 8.3, 50 mM KCl, 1.5 mM MgCl_2_, 200 μM dNTP mix, 0.2 μM each primer, and 1 U Taq DNA polymerase. Relative expression levels were normalized to glyceraldehyde-3-phosphate dehydrogenase (GAPDH) and calculated using the 2^–ΔCCt^ method. All PCR primers have been described previously [45,46].

### VEGF concentration measurement

To measure the concentration of VEGF secreted by ADSCs, 1.5 × 10^6^ viable ADSCs were seeded in 75 cm^2^ culture flasks containing DMEM/F12 with 10% PRP, 15% PRP, 20% PRP, or 10% FBS. These cells were incubated at 37°C with 5% CO_2_ for 72 hours. The media were then replaced, and the cells were incubated for a further 72 hours. The culture supernatants were collected, centrifuged at 4,980 × *g* for 10 minutes and stored at −80°C until use. The concentration of VEGF was then determined by an ELISA kit (Abcam, Cambridge, MA, USA). VEGF concentrations were also measured in the fresh media. VEGF produced by ADSCs was calculated by subtracting the values in culture supernatants from those in the fresh media.

### Stem cell transplantation

To evaluate the effects of PRP on ADSC transplantation in osteoarthritis, we used a mouse model of articular cartilage injury. In this experiment, we compared the efficiency of transplantation using ADSCs treated with 15% PRP (PRP15 group) or 10% FBS (FBS10 group), and control PBS injection. All procedures were approved by the Local Ethics Committee of the Stem Cell Research and Application Laboratory, University of Science. Articular cartilage injury was induced by joint destruction in the hind limbs of NOD/SCID mice using a 32 G needle. Briefly, 12 mice were anesthetized using ketamine (40 mg/kg) and then subjected to hind-limb joint destruction. An uninjured mouse was used as a control. Injured mice were equally divided into the PRP15 group (four mice), in which mice were transplanted with ADSCs cultured with 15% PRP; the FBS10 group (four mice), in which mice were transplanted with ADSCs cultured with 10% FBS; and the negative control group (four mice), in which mice were injected with PBS. The mice were then anesthetized and injected with either ADSCs or PBS (negative control). In the treatment groups, 2 × 10^6^ ADSCs of the PRP15 or FBS10 groups suspended in 200 μl PRP were injected into the knee joint via two doses with a 10-minute interval between injections.

For functional evaluation, hind-limb movement was then evaluated daily. Mice were placed in water. The natural response was a pedal response in water. We recorded the pedal response of treated hind limbs. After 45 days, all mice were euthanized and their hind limbs were used for histological analysis and further experiments. The samples were fixed in 10% formalin, decalcified, sectioned longitudinally, and stained with H & E (Sigma-Aldrich). Using H & E-stained sections, three parameters were examined for the knee joints: the area of damaged cartilage (%), the area of regenerated cartilage (%), and the number of regenerated cartilage cell layers. The damaged cartilage area was determined by mature cartilage that was lost compared with that in the control.

### Statistical analysis

All experiments were performed in triplicate. *P* ≤0.05 was considered significant. Data were analyzed using Statgraphics software 7.0 (Statgraphics Graphics System, Warrenton, VA, USA).

## Results

### ADSCs proliferate *in vitro* and maintain expression of specific markers after several passages

**Figure 1:**
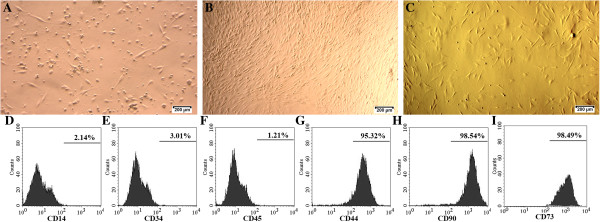
**Adipose-derived stem cell culture and marker confirmation. (A)** At 24 hours after seeding, fibroblast-like cells adhered to the surface of the flask, **(B)** proliferated and reached confluence after 1 week, and **(C)** became homogeneous after three subcultures. At the third passage, adipose-derived stem cells expressed mesenchymal stem cell-specific markers including **(G)** CD44, **(H)** CD90, and **(I)** CD73, while **(D)** CD14, **(E)** CD34, and **(F)** CD45 were negative.

We successfully isolated the SVF from adipose tissue. A total of 1.43 ± 0.15 × 10^6^ stromal cells with a viability of 94.4 ± 3.54% were collected from 1 g adipose tissue (*n* = 10). The cells were cultured with a 100% success rate (10/10) without microorganism contamination. After 24 hours of incubation, fibroblast-like cells appeared in the cultures (Figure 1A). From day 3, cells rapidly proliferated and reached confluence on day 7 (Figure 1B). The cells were subcultured three times before use in experiments. After the third passage, the cells maintained a homogeneous fibroblastic shape (Figure 1C).

The cells expressed MSC-specific markers with >95% positive staining for CD44, CD73, and CD90 (Figure 1G, H, I), and <4% of cells were positive for hematopoietic markers CD14, CD34 and CD45 (Figure 1D, E, F). Moreover, they also hold potential differentiation into specific cells. In fact, they were successfully differentiated into adipocytes in previous published research [47]. These cells were considered to be ADSCs and used for further experiments.

### Platelet-rich plasma efficiently stimulates ADSC proliferation

**Figure 2:**
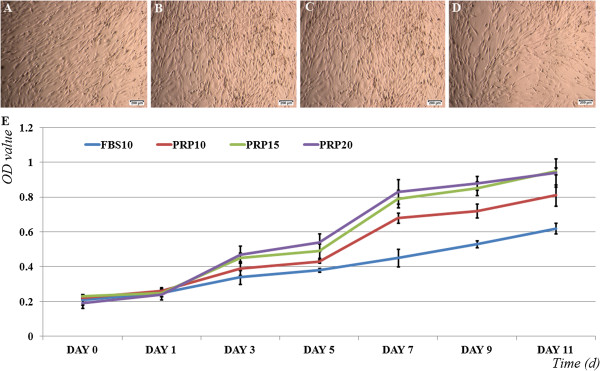
**Adipose-derived stem cell proliferation in experimental groups.** Adipose-derived stem cells (ADSCs) maintained the shape in four different media: **(A)** 10% platelet-rich plasma (PRP10), **(B)** 15% PRP (PRP15), **(C)** 20% PRP **(**PRP20) and **(D)** 10% fetal bovine serum (FBS10). **(E)** ADSC proliferation significantly increased in medium containing PRP at 10%, 15%, and 20% compared with that in medium containing 10% FBS. OD, optical density.

To investigate the effects of PRP on ADSC proliferation, we performed cell proliferation assays. The results showed that PRP could replace FBS in growth medium. In the mice transplanted with ADSCs cultured with 10% PRP (PRP10 group), in the PRP15 group, and in the mice transplanted with ADSCs cultured with 20% PRP (PRP20 group), ADSCs adhered to the flask surface. Under a microscope, ADSCs exhibited a normal shape (Figure 2A, B, C) similar to that of FBS-cultured ADSCs (Figure 2D). In MTT assays, we found that PRP strongly stimulated ADSC proliferation. At the three concentrations of PRP, ADSC proliferation was stimulated more strongly than that in medium containing 10% FBS (FBS10 group). After 3 days of PRP treatment, ADSCs started to increase their proliferation rate compared with that in the control (FBS10 group). The differences were statistically significant at day 7 in all three groups treated with PRP (Figure 2E). Compared with 10% PRP and 10% FBS, 15% PRP and 20% PRP stimulated ADSC proliferation more strongly. However, the difference between 15% PRP and 20% PRP was not significant. We therefore concluded that 15% PRP was the optimal concentration for robust proliferation of ADSCs.

### Platelet-rich plasma does not change marker expression but induces expression of genes related to chondrocytes

**Figure 3:**
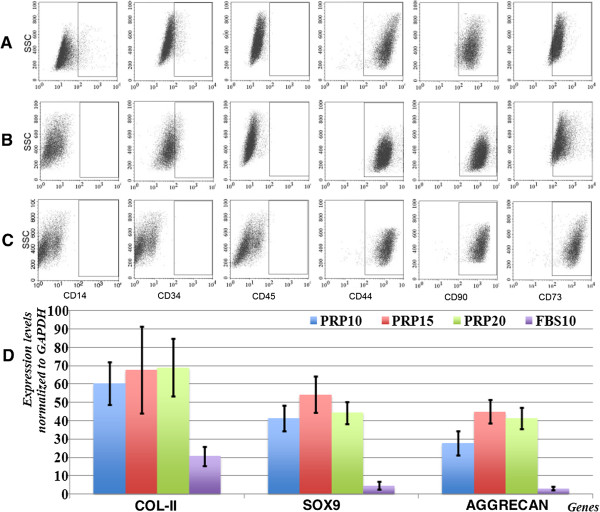
**Platelet-rich plasma does not change adipose-derived stem cell marker expression but changes chondrocyte-related gene expression.** The expression of CD14, CD34, CD44, CD45, CD73, and CD90 was changed in the **(A)** 10% platelet-rich plasma (PRP10), **(B)** 15% PRP (PRP15), and **(C)** 20% PRP (PRP20) groups compared with the 10% fetal bovine serum (FBS10) group (Figure 1). **(D)** Expression of collagen type II (COL-II), Sox9, and aggrecan was strongly promoted in the PRP10, PRP15, and PRP20 groups compared with that in the FBS10 group. GAPDH, glyceraldehyde-3-phosphate dehydrogenase; SSC.

Figure 3 shows the percentages of ADSCs expressing specific markers in the three groups. The percentages of ADSCs expressing CD44, CD73, and CD90 were 98.32 ± 1.21%, 97.21 ± 3.21%, and 96.21 ± 1.22% for CD44, 95.12 ± 2.12%, 96.27 ± 2.19%, and 95.54 ± 3.10% for CD73, 98.81 ± 1.11%, 97.37 ± 1.27%, and 98.92 ± 2.01% for CD90 in the PRP10, PRP15, and PRP20 groups, respectively. The percentages of ADSCs expressing CD14, CD34, and CD45 were 2.13 ± 1.11%, 2.65 ± 1.21%, and 1.98 ± 0.45% for CD14, 0.21 ± 0.11%, 0.98 ± 0.09%, and 1.31 ± 0.89% for CD34, and 2.11 ± 0.87%, 1.63 ± 1.08%, and 1.55 ± 0.51% for CD45 in the PRP10, PRP15, and PRP20 groups, respectively (Figure 3A, B, C). Compared with FBS (Figure 1D, E, F, G, H, I), these results showed that the three concentrations of PRP did not affect marker expression of ADSCs.

However, there were differences in the expression of some genes including col-II, Sox9, and aggrecan. Compared with the FBS10 group, ADSCs in the PRP10, PRP15 and PRP20 groups showed increased expression of col-II, Sox9, and aggrecan, all of which are important for chondrogenesis. As shown in Figure 3D, col-II expression increased from 20.07 ± 5.13 (compared with GAPDH) to 60.33 ± 11.68, 67.67 ± 23.80, and 69.00 ± 15.62 in the FBS10, PRP10, PRP15, and PRP20 groups, respectively (*P* ≤0.05). Similarly, expression of chondrogenic markers Sox9 and aggrecan also increased in the PRP10, PRP15, PRP20 groups compared with that in the FBS10 group. Sox9 expression increased from 4.67 ± 2.08 in the FBS10 group to 41.33 ± 7.09, 54.33 ± 10.07, and 44.33 ± 6.03 (compared with GAPDH) in the PRP10, PRP15, and PRP20 groups, respectively (*P* ≤0.05). Aggrecan expression also increased from 3.00 ± 1.00 in the FBS10 group to 27.67 ± 6.51, 45.00 ± 6.24, and 41.33 ± 5.86 in the PRP10, PRP15 and PRP20 groups, respectively (*P* ≤0.05). These data demonstrated that PRP changed the gene expression of ADSCs toward the chondrogenic lineage but did not change the surface marker expression of ADSCs.

### Platelet-rich plasma-treated ADSCs secrete less VEGF-A

**Figure 4:**
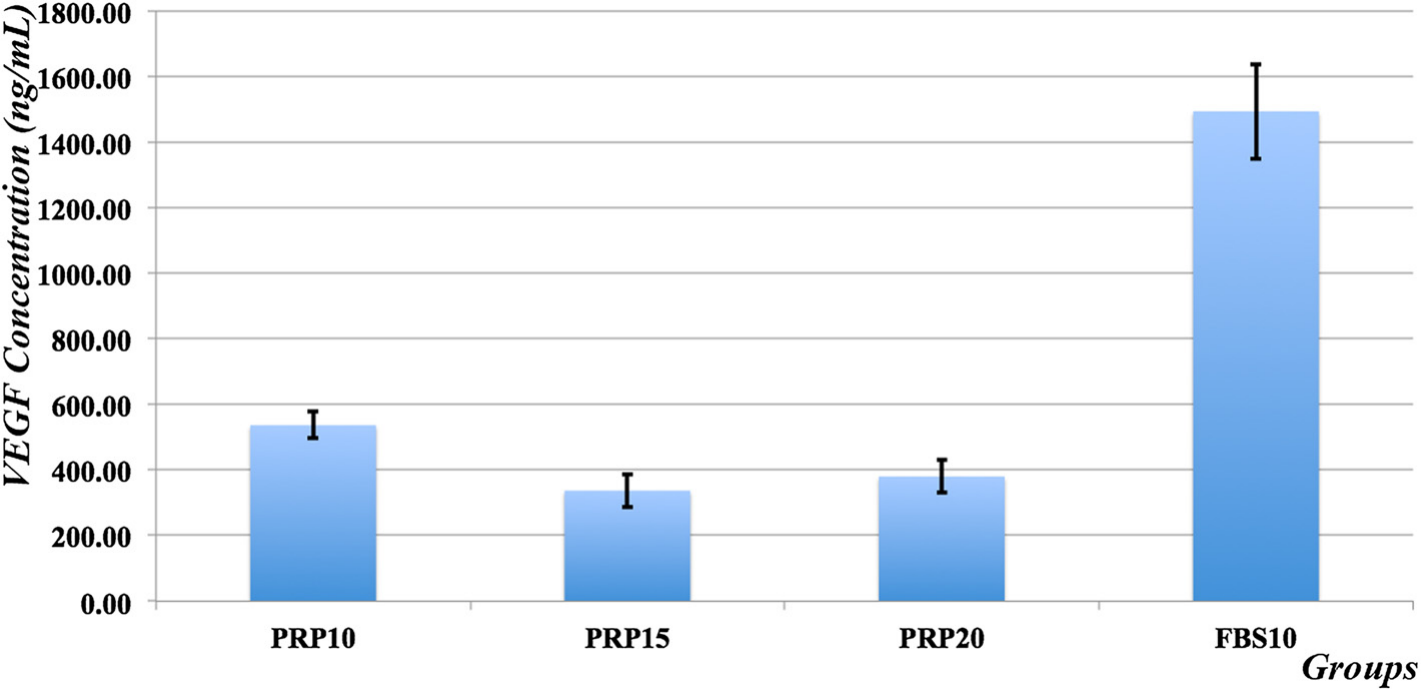
**Vascular endothelial growth factor-A secretion is reduced in platelet-rich plasma-treated adipose-derived stem cells.** Vascular endothelial growth factor (VEGF)-A concentrations were significantly decreased in culture supernatants of the 10% platelet-rich plasma (PRP10), 15% PRP (PRP15), and 20% PRP (PRP20) groups compared with that in the 10% fetal bovine serum (FBS10) group.

The results showed that ADSCs in the PRP10, PRP15, and PRP20 groups produce less VEGF-A. The concentrations of VEGF were 536.67 ± 40.41 ng/ml, 336.67 ± 51.32 ng/ml, 380.0 ± 50 ng/ml, and 1,493.33 ± 143.64 ng/ml in the PRP10, PRP15, PRP20, and FBS10 groups, respectively (Figure 4). Compared with the FBS10 group, these decreases were significant in the PRP10, PRP15, and PRP20 groups. VEGF concentrations in the PRP15 and PRP20 groups significantly decreased compared with that in the PRP10 group, indicating that VEGF expression was inhibited more efficiently at higher concentrations of PRP. However, the reduction of VEGF was not significant when increasing the concentration of PRP from 15% to 20%. Taken together, PRP decreased VEGF-A expression by 2.78-fold, 4.44-fold, and 3.93-fold in the PRP10, PRP15, PRP20 groups compared with that in the FBS10 group, respectively. This result suggests that transplantation of PRP-treated ADSCs may improve injured articular cartilage.

### Articular cartilage regeneration by platelet-rich plasma-treated ADSC transplantation

**Figure 5:**
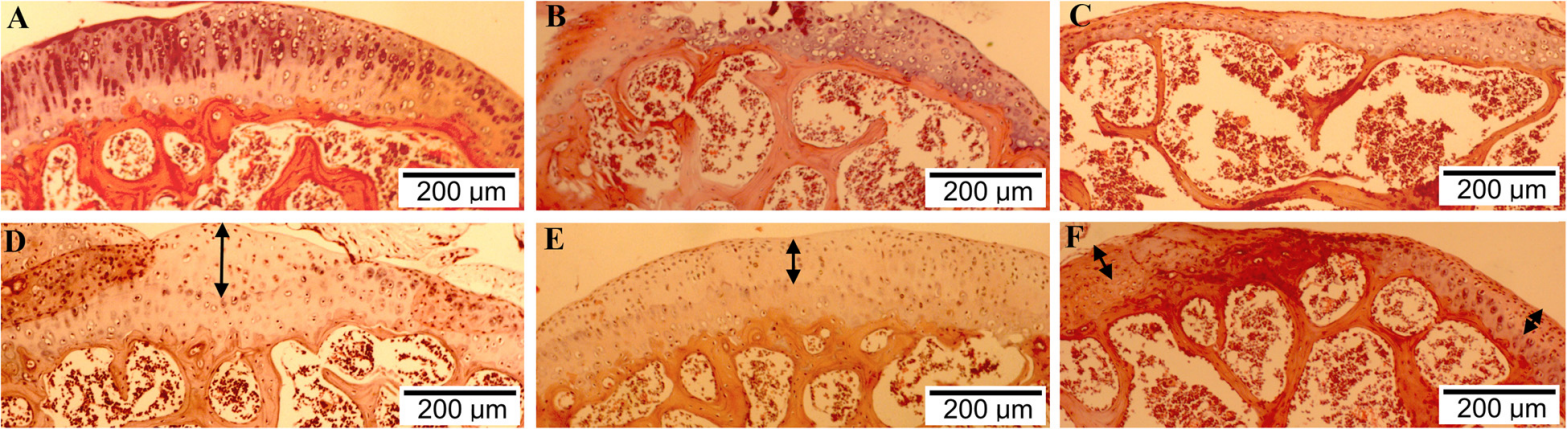
**Recovery of mouse knee joints. (A)** The cartilage layer of 15% platelet-rich plasma (PRP)-cultured adipose-derived stem cell (ADSC)-treated mice was similar to that in normal mice. There was evidence of regenerated cartilage formation at the articular cartilage margin in the treated mice, and the thickness of the cartilage layer of the treated mice compared with **(B)** before treatment and **(C)** control. H & E-stained articular cartilage sections of mice that received **(D, E)** 15% PRP-cultured ADSC transplantation, **(F)** 10% fetal bovine serum (FBS)-cultured ADSC transplantation, or **(C)** PBS injections.

The results showed a significant difference among the treatment and negative control groups, especially in terms of the time until mice could control their hind-limb movement as well as regeneration of the joint cartilage. The time until recovery of hind-limb movement decreased from 32.5 ± 7.5 days in negative control (PBS-injected) mice to 17.5 ± 3.5 days in the PRP15 group, but did not decrease for the FBS10 group (30.5 ± 5.5 days). In the PRP15 mice, histological analysis showed that the mean area of damaged joint cartilage was 70% with 45% of regenerated cartilage formed after 45 days. This regenerated cartilage layer had about 12 layers of chondrocytes. However, in mice of the FBS10 group the mean area of damaged joint cartilage was 70%, but there was only 30% regenerated cartilage formed after 45 days and about eight layers of chondrocytes. In the negative control mice, the mean area of damaged joint cartilage was 80%, but there was only 20% regenerated cartilage formed after 45 days and five layers of chondrocytes (Figure 5).

## Discussion

PRP is a natural source of growth factors. In this study, we determined the effects of PRP on ADSC transplantation in an injured articular cartilage model. To investigate the physiological changes of ADSCs induced by PRP, we successfully isolated ADSCs and PRP.

We isolated the SVF with good viability from adipose tissue. From the SVF, we isolated ADSCs that expressed some MSC characteristics including expression of CD44, CD74, and CD90, and the absence of hematopoietic cell lineage markers CD14, CD34, and CD45. These cells differentiated into adipocytes *in vitro*. We also prepared PRP with growth factors enriched by five to seven times compared with those in normal plasma (data not shown).

Next, we evaluated the effects of PRP on ADSC proliferation. The results from MTT assays showed that PRP strongly stimulated ADSC proliferation, demonstrating that PRP contains growth factors that are essential for ADSC proliferation. There are numerous important growth factors, such as basic fibroblast growth factor (bFGF), epidermal growth factor, and platelet-derived growth factor, which stimulate stem cell proliferation [48,49]. In previous studies, PRP efficiently stimulated ADSC proliferation [50-53]. Kocaoemer and colleagues showed that ADSCs rapidly proliferate in medium supplemented with 10% human serum and 10% PRP rather than 10% FBS [50]. However, in contrast to our results showing that 15% PRP was the optimal concentration in medium to stimulate proliferation, Kakudo and colleagues showed that 5% activated PRP maximally promotes ADSC proliferation, whereas 20% activated PRP does not promote proliferation [53]. More importantly, PRP not only stimulates ADSC proliferation but also preserves the differentiation potential of ADSC *in vitro*[51,52]. However, Gharibi and Hughes recently showed that ADSCs treated with bFGF, epidermal growth factor, platelet-derived growth factor, and ascorbic acid show a loss of differentiation potential prior to reaching senescence [48], indicating that PRP may induce differentiation into functional cells.

In our study, we considered that PRP not only stimulates ADSC proliferation but also differentiation into chondrogenic cells. We therefore investigated the changes of ADSC phenotype when cultured in medium supplemented with PRP or FBS. PRP did not change surface marker expression of ADSCs after culture in PRP-containing medium for 1 week. However, there were significant differences in the expression of chondrogenesis-related genes.

ADSCs treated with PRP exhibited upregulated expression of chondrogenesis-related gene such as col-II, Sox9, and aggrecan. We found that col-II gene expression increased by 3.01-fold, 3.37-fold, and 3.44-fold in the PRP10, PRP15, and PRP20 groups, compared with that in the FBS10 group, respectively. Similarly, expression of other chondrogenic markers including Sox9 and aggrecan also increased in the PRP10, PRP15, and PRP20 groups compared with that in the FBS10 group. Sox9 expression strongly increased in the PRP10, PRP15 and PRP20 groups compared with that in the FBS10 group. These results demonstrated that PRP changed the gene expression of ADSCs toward the chondrogenic lineage but did not change the surface marker expression of ADSCs.

The secretion of certain growth factors, especially VEGF-A from ADSCs, inhibits cartilage regeneration [43]. VEGF enhances catabolic pathways in chondrocytes, and VEGF overexpression is associated with progression of osteoarthritis in articular cartilage [54,55]. In fact, VEGF induces matrix metalloproteinase expression in immortalized chondrocytes [56]. We therefore considered that PRP may not only promote ADSC differentiation into chondrogenic cells but might also inhibit VEGF secretion. For this reason, PRP-treated ADSCs may induce chondrocyte differentiation and regenerate cartilage. We confirmed that, after treatment with PRP for 1 week, ADSCs downregulated VEGF secretion into the culture supernatant. PRP10, PRP15 and PRP20 ADSCs downregulated VEGF expression by 2.78-fold, 4.44-fold, and 3.93-fold compared with that in FBS10 ADSCs, respectively. This observation indicates that PRP-treated ADSCs may improve ADSC transplantation in injured articular cartilage. In fact, Lee and colleagues improved ADSC transplantation in cartilage regeneration by neutralizing VEGF with mAbs [43] .

PRP showed several beneficial effects on ADSCs for chondrogenic differentiation *in vitro*. Similarly, in muscle-derived stem cells, PRP promotes the expression of bone morphogenic protein-4, promotes collagen synthesis, suppresses chondrocyte apoptosis, and enhances the integration of transplanted cells in the repair process [57]. PRP also increases cartilage catabolism in synoviocytes [58]. The effects of PRP are induced by growth factors of the platelets. As indicated above, PRP contains several important growth factors that have effects on proliferation and differentiation, such as bFGF and transforming growth factor-beta. In fact, bFGF enhances the kinetics of MSC chondrogenesis, leading to early differentiation, possibly by a priming mechanism [59]. In addition, bFGF induces ADSC chondrogenesis [60,61]. bFGF-treated bone marrow-derived MSCs also undergo chondrogenic differentiation [62]. Furthermore, transforming growth factor-beta stimulates chondrogenic differentiation of MSCs [63,64].

We also evaluated the role of PRP in chondrogenesis *in vivo*. The results showed significantly different efficiencies of injured articular regeneration by transplantation of PRP-treated (PRP15 group) and untreated ADSCs (FBS10 group). PRP15 ADSC transplantation efficiently reduced the recovery time of hind-limb movement compared with that of ADSC transplantation alone. Importantly, ADSC transplantation showed an effect compared with that of the control (PBS injection), but not significantly. Stimulation of cartilage regeneration was also achieved in PRP15 ADSC transplantation. Compared with FBS10 ADSC transplantation and PBS injection, PRP15 ADSCs efficiently stimulated cartilage formation. ADSC transplantation also stimulated cartilage formation compared with that of PBS injection but more slowly and at a lower efficiency. These results showed that PRP is an important factor that promotes both *in vitro* and *in vivo* chondrogenesis of ADSCs. Previous studies have performed co-transplantation of ADSCs and PRP in dogs [30-32,35], and co-transplantation of the SVF and PRP in humans [41,42,65] and mice [37,38], resulting in significant improvements of injured articular cartilage. Transplantation of ADSCs without PRP in rats [43] or SVF transplantation without PRP in horses [34] inhibits cartilage regeneration [43] or provides insignificant improvements [34].

## Conclusion

Adipose tissue provides a rich source of MSCs. ADSCs have been used to treat injured articular cartilage in recent years. However, ADSC transplantation in injured articular cartilage has caused controversy regarding the treatment efficiency and ADSC transplantation combined with additional factors to induce chondrogenic differentiation. This study revealed that PRP is a suitable factor in ADSC transplantation to treat injured articular cartilage. PRP stimulates ADSC proliferation and induces ADSC differentiation into chondrogenic cells with overexpression of col-II, Sox9, and aggrecan. In particular, PRP reduces VEGF expression that inhibits cartilage regeneration to improve cartilage regeneration *in vivo* by PRP-treated ADSC transplantation. PRP-treated ADSC transplantation significantly improves cartilage formation in murine models compared with that of untreated ADSC transplantation. These results reveal a promising therapy of injured articular cartilage by transplantation of ADSCs combined with PRP.

## Abbreviations

ADSC: Adipose-derived stem cell; bFGF: Basic fibroblast growth factor; BSA: Bovine serum albumin; col-II: Type II collagen; DMEM: Dulbecco’s modified Eagle’s medium; ELISA: Enzyme-linked immunosorbent assay; FBS: Fetal bovine serum; GAPDH: Glyceraldehyde-3-phosphate dehydrogenase; H & E: Hematoxylin and eosin; mAb: Monoclonal antibody; MSC: Mesenchymal stem cell; PBS: Phosphate-buffered saline; PCR: Polymerase chain reaction; PRP: Platelet-rich plasma; RT: Reverse transcriptase; SVF: Stromal vascular fraction; VEGF: Vascular endothelial growth factor.

## Competing interests

The authors declare that they have no competing interests.

## Authors’ contributions

PVP carried out studies including primary culture, ADSC isolation and culture, PRP preparation, and manuscript writing. KH-TB, TDD, TDN, and VTL collected the adipose tissue and peripheral blood, and established animal models. DQN carried out the histological analysis of cartilage. NBV, NHT performed the stem cell transplantation in murine models, and evaluated injured articular cartilage healing. DML and NL-CP performed gene expression analyses and measured the VEGF-A concentrations. NKP revised the manuscript, edited figures, and processed data. All authors read and approved the final manuscript.
